# Supplementary material for: Discovery of driver non-coding splice-site-creating mutations in cancer
Source: Nat Commun. 2020 Nov 4;11:5573. doi: 10.1038/s41467-020-19307-6 (PMC7642382; doi:10.1038/s41467-020-19307-6)
Supplement: Supplementary file 1 — Supplementary Information [file 41467_2020_19307_MOESM1_ESM.pdf]

## Supplementary Table 1: Primers used in this study

| Gene | Primer Type | Forward Primer | Reverse Primer |
|---|---|---|---|
| EP300 | Genomic Primer | TTAAGGATCCAGAAGGAAACCACAGGCTCAC | AATTACGCGTGTGGACCCAATGGCTTAGCTT |
| EP300 | Q5 Mutagenesis | TGTGACCTGACTTTTTTTTTCCTCTTCATTTCTCTTCAGTTTGTAT | TAATGAGTTCTTTACTCCATGTGCCTAATAACACCCT |
| BCOR | Genomic Primer | TTAAGGATCCGAGAATGTCTGTCTGTATGCAATAGT | AATTACGCGTGGAGATGGCTGGGAAGTGTC |
| BCOR | Q5 Mutagenesis | AACGAAAGTGAGAGATGTTAGTAAACCTG | CTGTTCTGCAATGGCCTCCT |
| DMNT3A | Genomic Primer | TATTGGATCCTGTGGCCTCCAGTGGTCTCCTTAG | AATTACGCGTTCTAAGTCCACGGACTGCATACGT |
| DMNT3A | Q5 Mutagenesis | TCACTGCTGAGGACCCTCC | GCACAACAGGTCAGATGCAG |
| KMT2B | Genomic Primer | TTAAGGATCCCCACGAGGTTAGATCTCTGCC | AATTACGCGTAGCCCCTAAAACCACTCCAT |
| KMT2B | Q5 Mutagenesis | CTCCACAGCCAACAGCACC | ACAGAGATGGCGAAACCTGC |
| MAP3K1 | Genomic Primer | TATAGGATCCAGTTAGCTACAACCAGGAGCC | TTAAACGCGTACTACCCATCCACCTCTCATCA |
| MAP3K1 | Q5 Mutagenesis | AAAGATTGCTTTAGTTTAATATG | TTACTGTTAAAGGAGTAAGA |

Primers for cDNA amplification,i.e., pCAS-KO1-(5′-TGACGTCGCCGCCCATCAC-3′) and pCAS-R (5′-ATTGGTTGTTGAGTTGGTTGTC-3′)

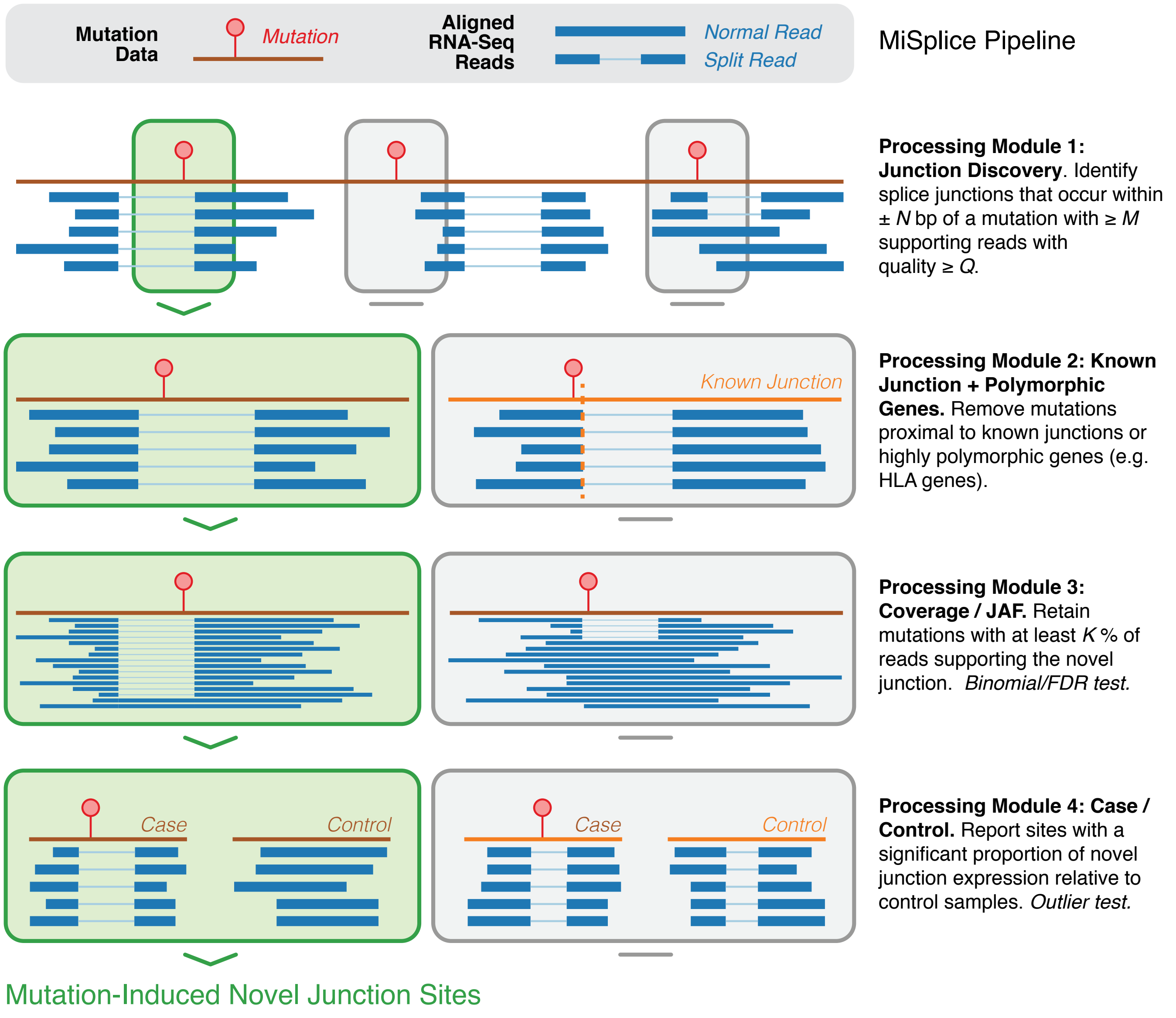


**Fig. S1: The workflow and schematic of the MiSplice pipeline.** Misplice pipeline includes four modules including mutation-induced junction discovery (Module 1), filtering known junction and polymorphic genes (Module 2), retain mutation-induced novel with sufficient supporting evidence (Modules 3 and 4).

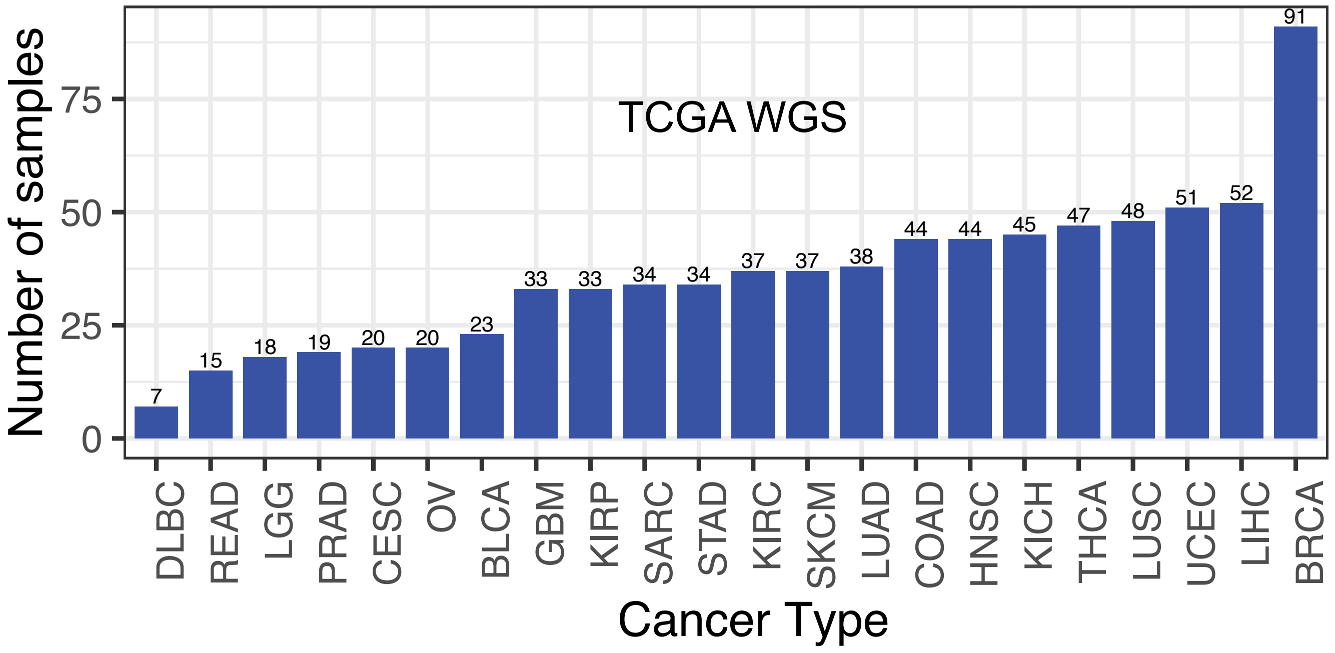


**Fig. S2: Sample set of WGS data.** The number of TCGA samples with WGS data and RNA-Seq across different cancer types. Source data are provided as a Source Data file.

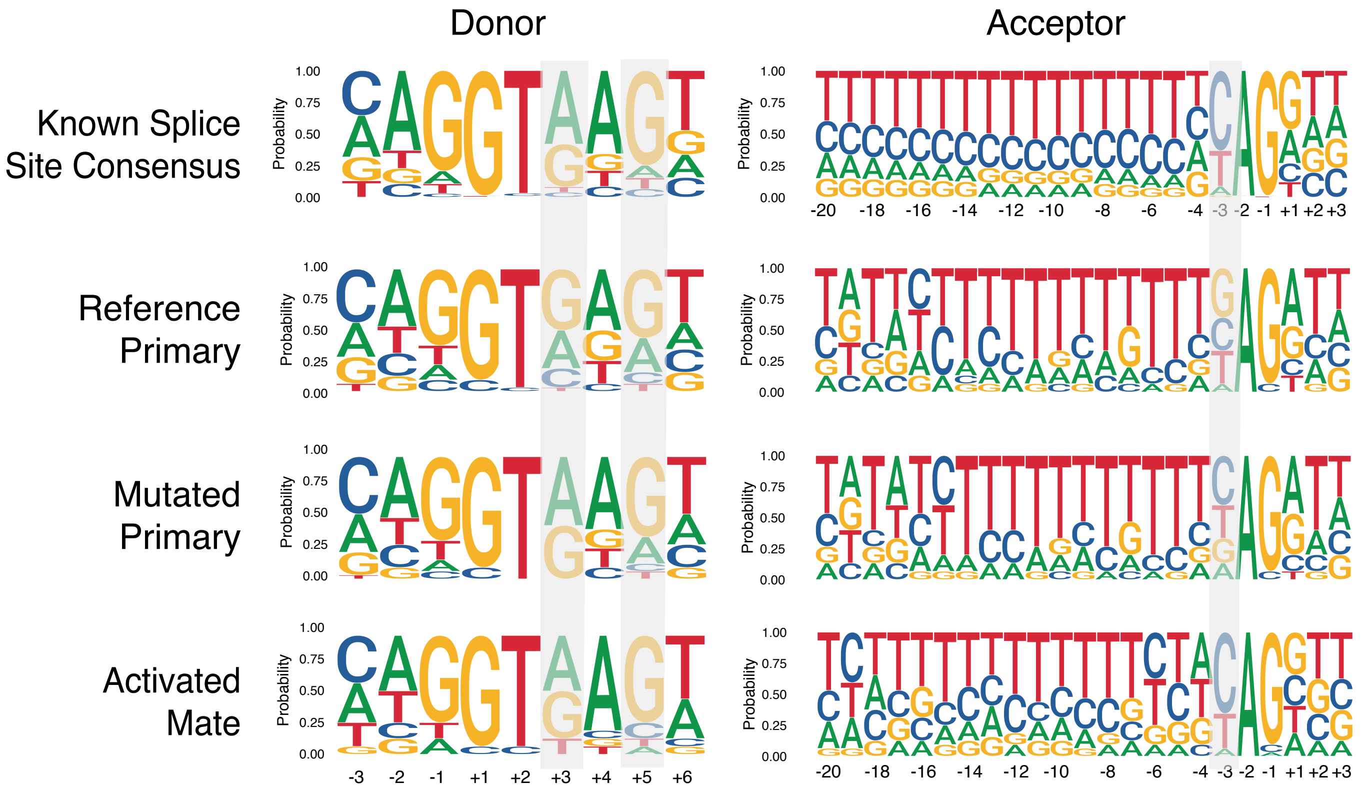


**Fig. S3: Comparison of consensus sequences in primary sites and activated sites of new exon splice sites to the known splice site consensus.** We highlighted the -3 positions of the acceptor site and the +3 and +5 positions of the donor site in gray. In these positions, the absolute probability difference of any single nucleotide component between mutated primary and reference primary is higher than 0.1. Source data are provided as a Source Data file.

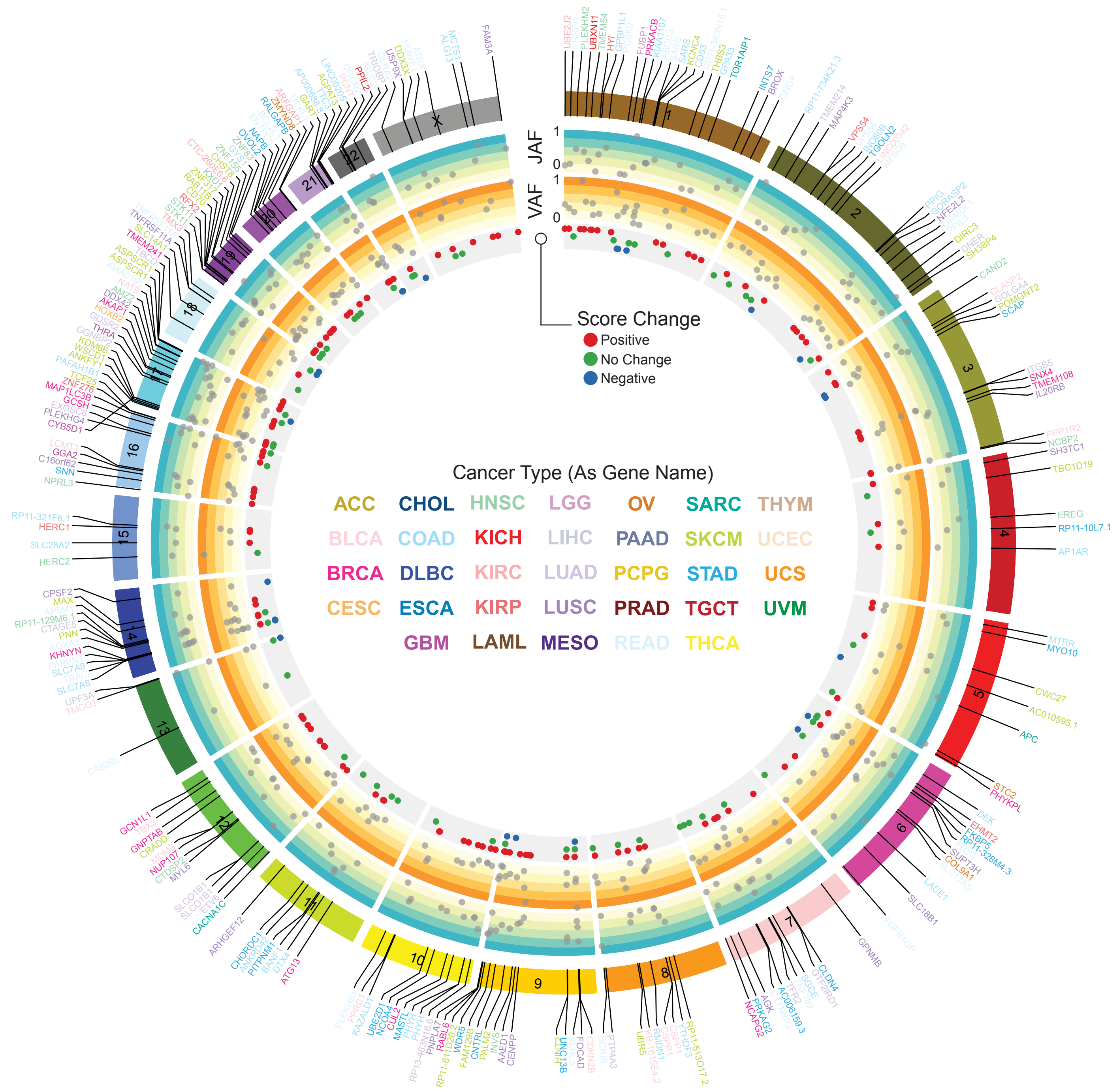


**Fig. S4: Circos plot of 228 non-coding splice-site-creating mutations.** From outer to inner rings: gene name, chromosome and mutation position, JAF, VAF, and change in splice score after mutation. Genes are colored by cancer types. Source data are provided as a Source Data file.

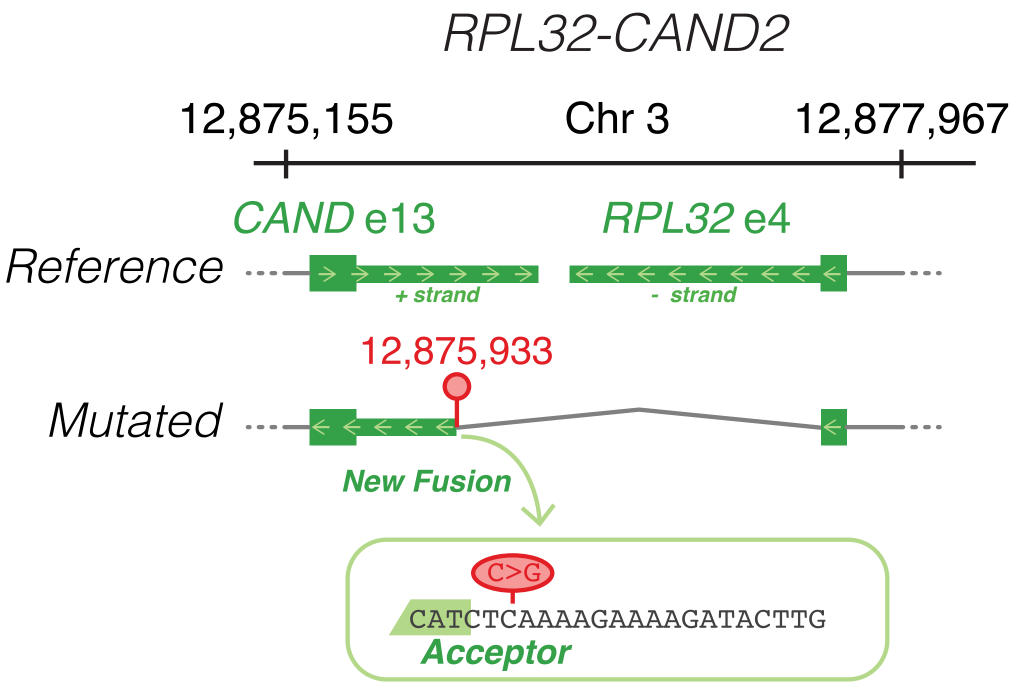


**Fig. S5: Schematics of RPL32-CAND2 fusion product.** A C>G CAND2 mutation at position 12,875,933 induces a splice site that leads to the fusion of the CAND2 UTR with the last coding exon of RPL32. Source data are provided as a Source Data file.

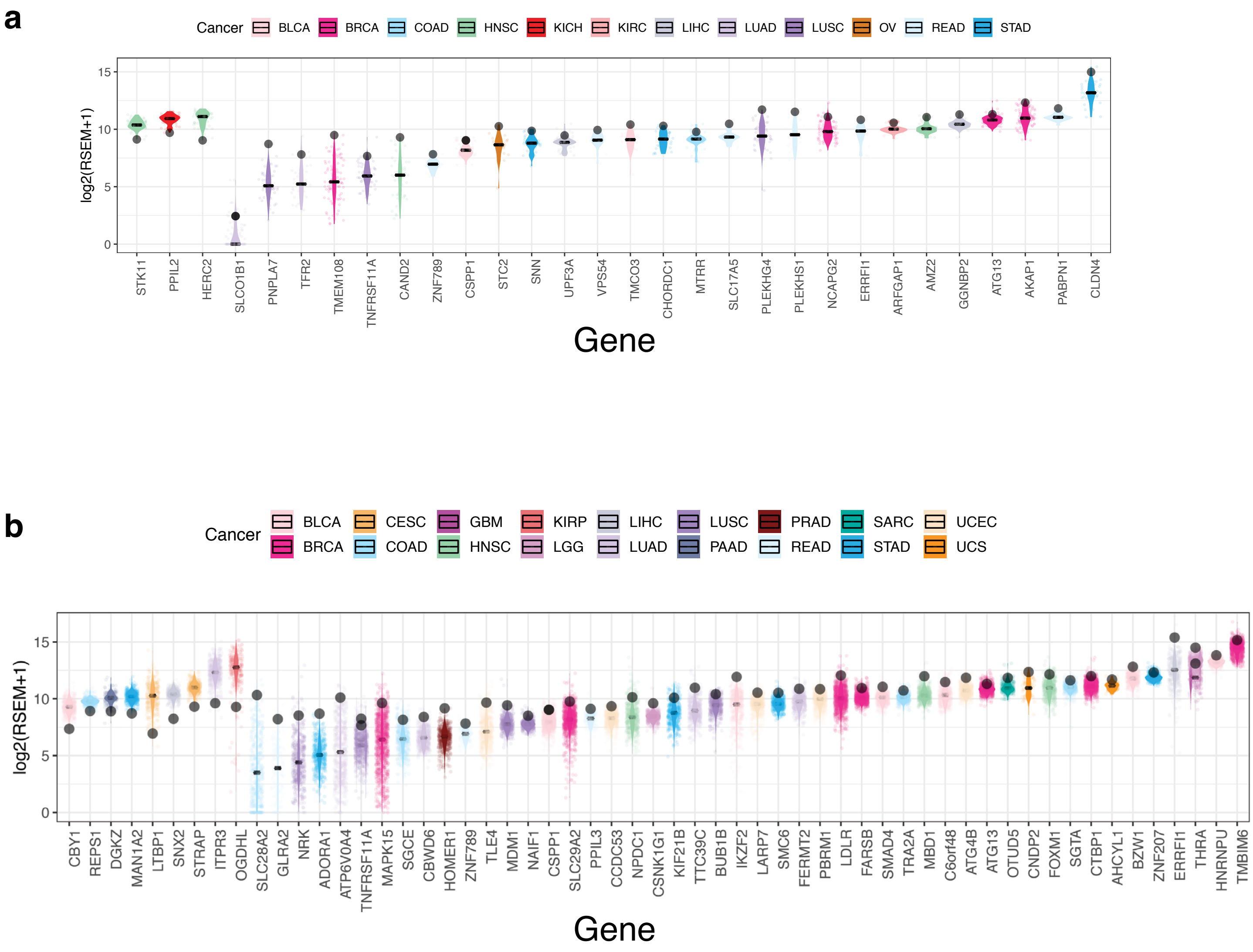


**Fig. S6: Expression outliers.** Expression outliers of non-coding splice-site-creating mutations (nc-SCMs) from a) TCGA WGS data and b) TCGA WXS data. The sample with nc-SCMs is marked as the black dot. The expression outlier analysis is at a per cancer type level. Source data are provided as a Source Data file.

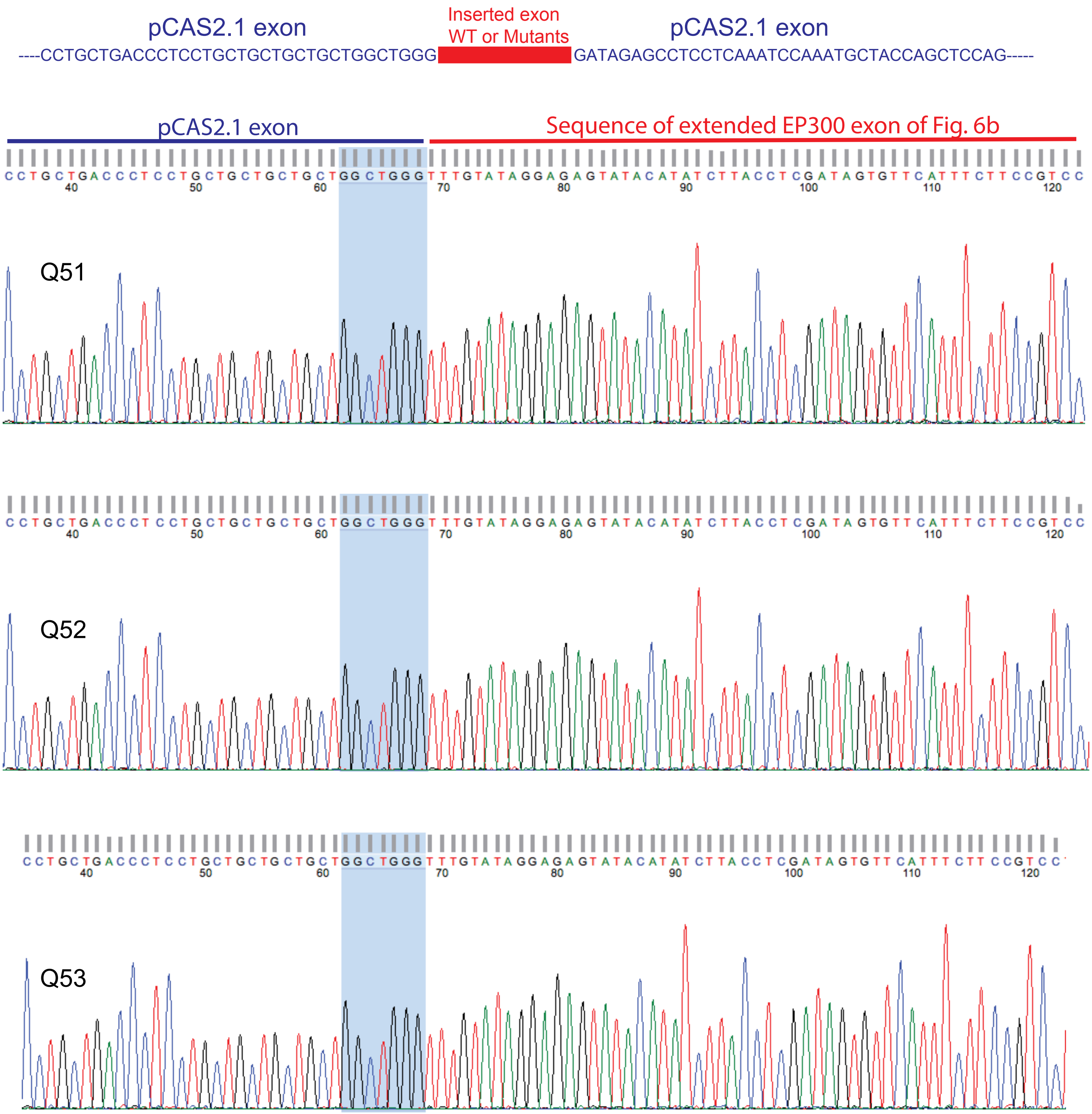


**Fig. S7: Sanger Sequencing of EP300 mutants.** Sanger sequencing confirmation of RT-PCR of three mutant triplicate of EP300. Sequences hightlighted indicates of boundary of pCAS2.1 exon.

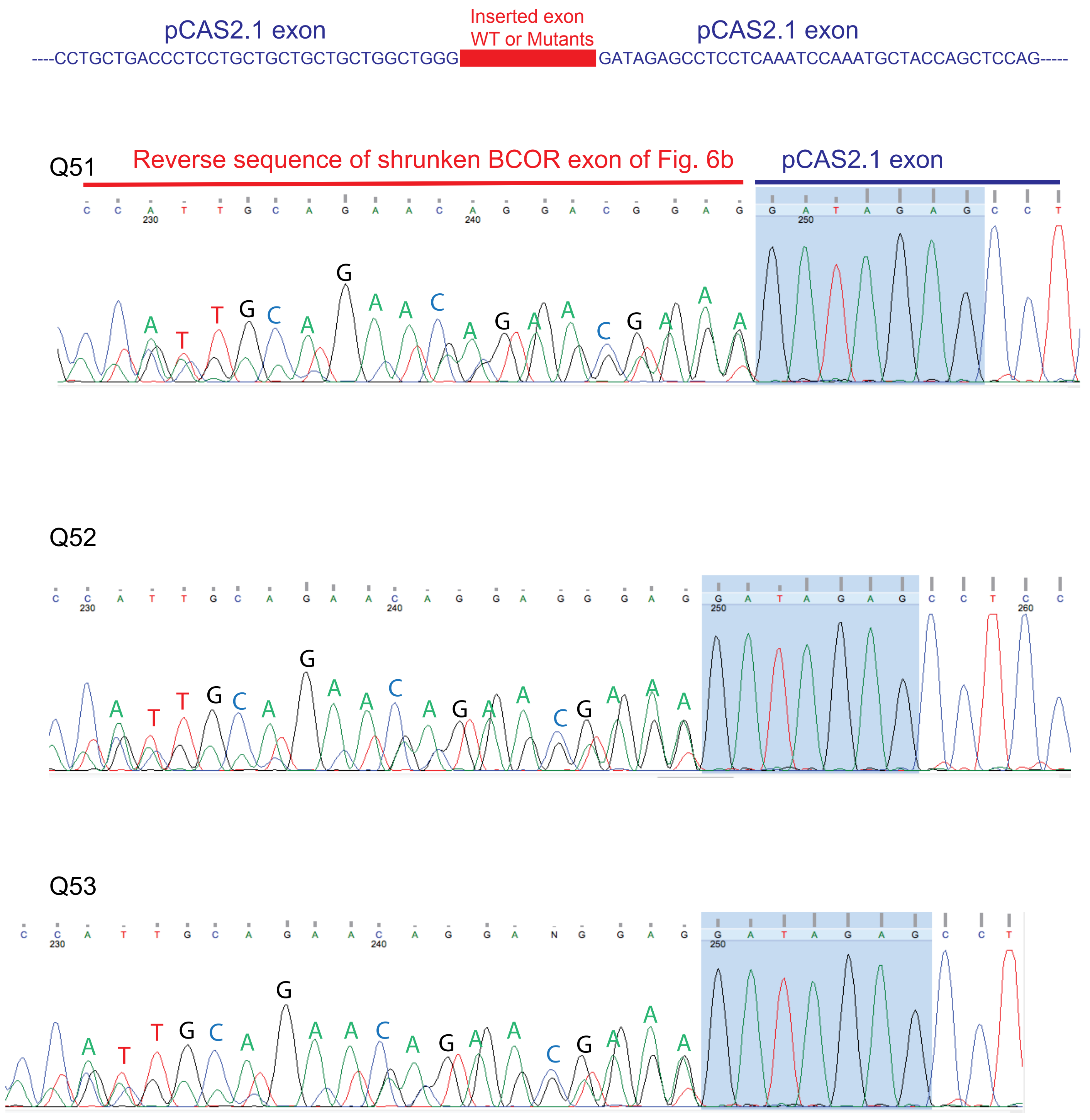


**Fig. S8: Sanger Sequencing of BCOR mutants.** Sanger sequencing confirmation of RT-PCR of three mutant triplicate of BCOR. Sequences hightlighted indicates of boundary of pCAS2.1 exon.

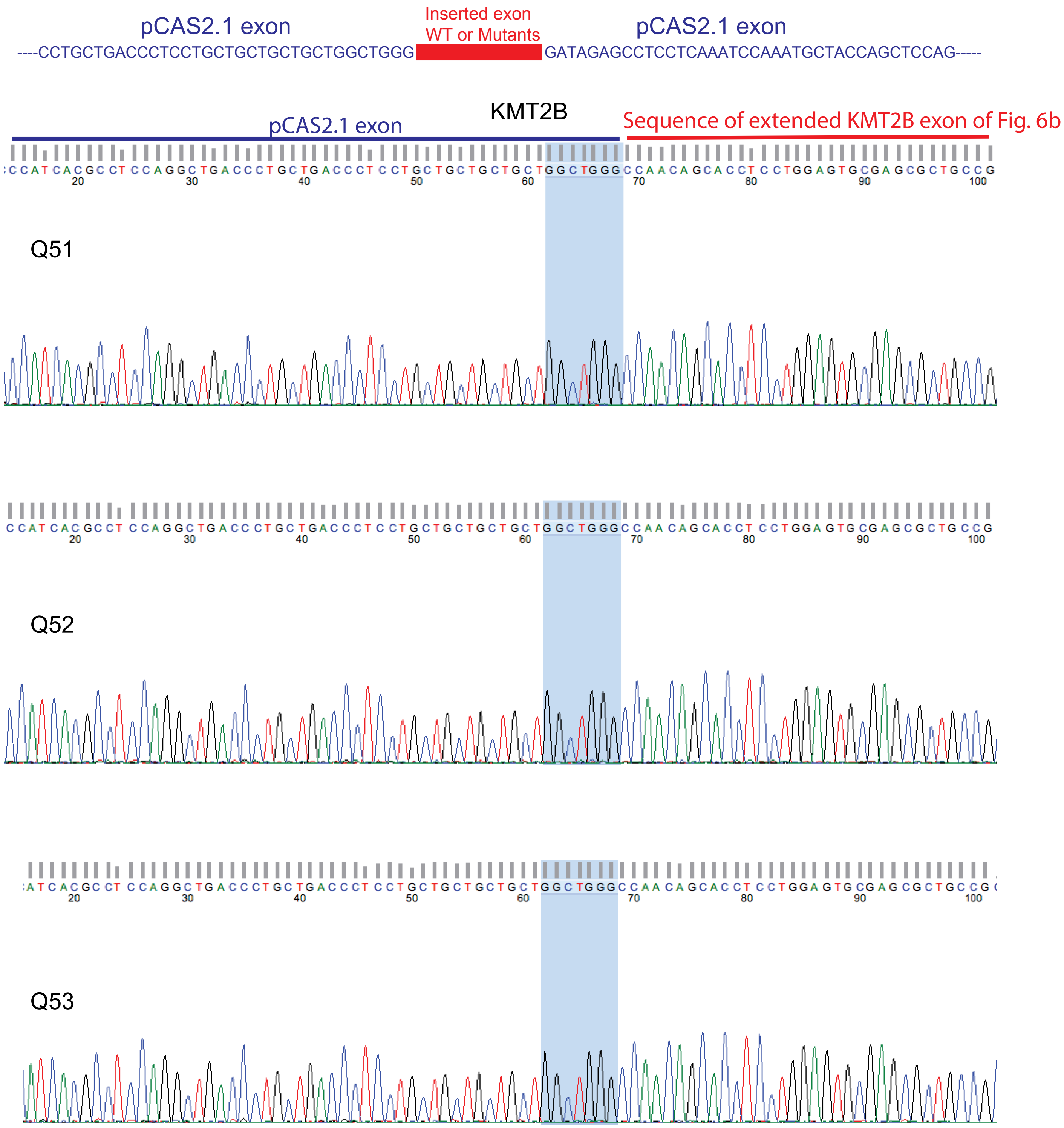


**Fig. S9: Sanger Sequencing of KMT2B mutants.** Sanger sequencing confirmation of RT-PCR of three mutant triplicate of KMT2B. Sequences hightlighted indicates of boundary of pCAS2.1 exon.

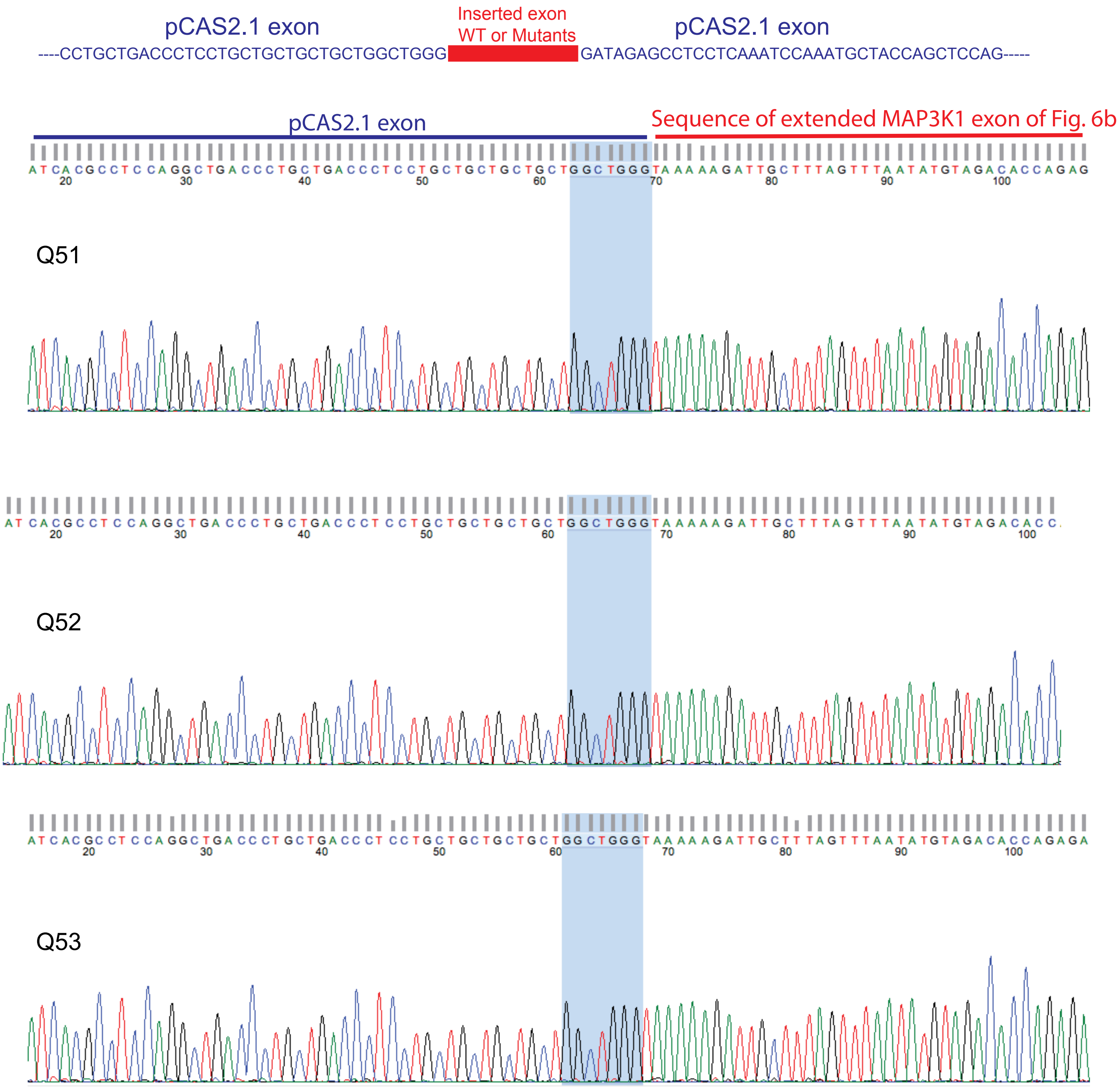


**Fig. S10: Sanger Sequencing of MAP3K1 mutants.** Sanger sequencing confirmation of RT-PCR of three mutant triplicate of MAP3K1. Sequences hightlighted indicates of boundary of pCAS2.1 exon.
