# Supplementary material for: Discovery of driver non-coding splice-site-creating mutations in cancer
Source: Nat Commun. 2020 Nov 4;11:5573. doi: 10.1038/s41467-020-19307-6 (PMC7642382; doi:10.1038/s41467-020-19307-6)
Supplement: Supplementary file 3 — Description of Additional Supplementary Files [file 41467_2020_19307_MOESM3_ESM.pdf]

Description of Additional Supplementary Files

File Name: Supplementary Data 1

Description: 228 non-coding splice-site-creating mutations found in 783 TCGA WGS data

File Name: Supplementary Data 2

Description: 369 non-coding splice-site-creating mutations found in 9,494 TCGA WXS data
