# Supplementary material for: Discovery of driver non-coding splice-site-creating mutations in cancer
Source: Nat Commun. 2020 Nov 4;11:5573. doi: 10.1038/s41467-020-19307-6 (PMC7642382; doi:10.1038/s41467-020-19307-6)
Supplement: Supplementary file 6 — Reporting Summary [file 41467_2020_19307_MOESM6_ESM.pdf]

nature research

Corresponding author(s): Li Ding

Last updated by author(s): Sep 23, 2020

Reporting Summary

Nature Research wishes to improve the reproducibility of the work that we publish. This form provides structure for consistency and transparency in reporting. For further information on Nature Research policies, see Authors & Referees and the Editorial Policy Checklist .

Statistics

For all statistical analyses, confirm that the following items are present in the figure legend, table legend, main text, or Methods section.

n/a	Confirmed	
☐	☒	The exact sample size (*n*) for each experimental group/condition, given as a discrete number and unit of measurement
☒	☐	A statement on whether measurements were taken from distinct samples or whether the same sample was measured repeatedly
☐	☒	The statistical test(s) used AND whether they are one- or two-sided<br>*Only common tests should be described solely by name; describe more complex techniques in the Methods section.*
☒	☐	A description of all covariates tested
☐	☒	A description of any assumptions or corrections, such as tests of normality and adjustment for multiple comparisons
☐	☒	A full description of the statistical parameters including central tendency (e.g. means) or other basic estimates (e.g. regression coefficient) AND variation (e.g. standard deviation) or associated estimates of uncertainty (e.g. confidence intervals)
☐	☒	For null hypothesis testing, the test statistic (e.g. *F*, *t*, *r*) with confidence intervals, effect sizes, degrees of freedom and *P* value noted<br>*Give P values as exact values whenever suitable.*
☒	☐	For Bayesian analysis, information on the choice of priors and Markov chain Monte Carlo settings
☒	☐	For hierarchical and complex designs, identification of the appropriate level for tests and full reporting of outcomes
☒	☐	Estimates of effect sizes (e.g. Cohen's *d*, Pearson's *r*), indicating how they were calculated

Our web collection on statistics for biologists contains articles on many of the points above.

Software and code

Policy information about availability of computer code

Data collection	no software was used for data collection.
Data analysis	MiSplice v1.2 used in current study is freely available at https://github.com/ding-lab/Misplice under the GNU general public license. Samtools v1.7 can be downloaded from https://github.com/samtools/samtools/releases/. MaxEntScan (no available version number) can be found from http://hollywood.mit.edu/burgelab/maxent/Xmaxentscan_scoreseq.html.

For manuscripts utilizing custom algorithms or software that are central to the research but not yet described in published literature, software must be made available to editors/reviewers. We strongly encourage code deposition in a community repository (e.g. GitHub). See the Nature Research guidelines for submitting code & software for further information.

Data

Policy information about availability of data

All manuscripts must include a data availability statement. This statement should provide the following information, where applicable:

- Accession codes, unique identifiers, or web links for publicly available datasets
- A list of figures that have associated raw data
- A description of any restrictions on data availability

The WGS mutation data for 790 TCGA samples with RNA-Seq were obtained from the International Cancer Genome Consortium (ICGC) at https://www.synapse.org/#!Synapse:syn7118450 (version 12-Oct-2016). We removed one outlier cancer type (DLBC) with only seven samples, which reduced the WGS samples set from 790 to 783; see supplementary Fig. 2. The full name of each cancer type included in the current study can be found at https://gdc.cancer.gov/resources-tcga-users/tcga-code-tables/tcga-study-abbreviations. The controlled-accessed WES mutation data were downloaded from GDC (https://gdc.cancer.gov/about-data/publications/mc3-2017). The ISB-CGC (https://isb-cgc.appspot.com) access of the TCGA RNA-seq bam corpus was granted through tcga-phs000178-controlled credential. The TCGA RNA-Seq alignments used in this study were generated by using MapSplice (https://academic.oup.com/nar/article/38/18/e178/1068935) against the hg19 reference genome. Details needed to replicate TCGA RNA-Seq bam file can be found at https://webshare.bioinf.unc.edu/public/mRNAseq_TCGA/. We also obtained gene expression data (RSEM) from the Broad firehose collection (http://gdac.broadinstitute.org/runs/stddata__2016_01_28/) across 33 TCGA cancer types.

Ensembl 37.75 database can be downloaded from ftp://ftp.ensembl.org/pub/release-75/gtf/homo_sapiens/. Source data underlying Figs. 1a-b, 2a-c, 3a-c, 4, 5a-b, 6a-b and Supplementary Figs 2-6 are provided as a Source Data file. All other data supporting the findings of this study are available from the corresponding author upon request.

Field-specific reporting

Please select the one below that is the best fit for your research. If you are not sure, read the appropriate sections before making your selection.

- [x] Life sciences
- [ ] Behavioural & social sciences
- [ ] Ecological, evolutionary & environmental sciences

For a reference copy of the document with all sections, see nature.com/documents/nr-reporting-summary-flat.pdf

Life sciences study design

All studies must disclose on these points even when the disclosure is negative.

Sample size	We selected all samples from TCGA and ICGC having both sequence and RNA-Seq data, namely 790 samples with both WGS mutation and RNA-Seq bam data, and 9,494 samples with both WES mutation and RNA-Seq bam data.
Data exclusions	We removed one outlier cancer type (DLBC) with only seven samples having both WGS mutation and RNA-Seq bam data. This is discussed in the Data Availability section.
Replication	We validated the sequence of mutation-induced splice junction through Sanger sequencing by three replicates.
Randomization	Discovery of npn-coding splice-site-creating mutations (nc-SCMs) is purely based on mutation status and RNA-Seq supporting evidence through MiSplice pipeline.
Blinding	The tumor data are provided by TCGA, and we are blind to the sample selection.

Reporting for specific materials, systems and methods

We require information from authors about some types of materials, experimental systems and methods used in many studies. Here, indicate whether each material, system or method listed is relevant to your study. If you are not sure if a list item applies to your research, read the appropriate section before selecting a response.

Materials & experimental systems

n/a	Involved in the study	
x		Antibodies
	x	Eukaryotic cell lines
x		Palaeontology
x		Animals and other organisms
x		Human research participants
x		Clinical data

Methods

n/a	Involved in the study	
x		ChIP-seq
x		Flow cytometry
x		MRI-based neuroimaging

Eukaryotic cell lines

Policy information about cell lines

Cell line source(s)	Cell line (HEK293T) is from ATCC.
Authentication	Cell line was authenticated by ATCC.
Mycoplasma contamination	The cell line was not tested for Mycoplasma contamination.
Commonly misidentified lines (See ICLAC register)	No commonly misidentified cell lines were used in the study.
